# Cardiac Arrest in the Airport Revealing Cocaine Body Packing: A Case Report

**DOI:** 10.1155/2019/6183154

**Published:** 2019-01-06

**Authors:** Dabor Resiere, Hossein Mehdaoui, Bruno Megarbane

**Affiliations:** ^1^Department of Medical and Toxicological Critical Care, Lariboisière Hospital, Paris-Diderot University, INSERM UMRS1144, Paris, France; ^2^Department of Medical Intensive Care Unit, University Hospital of Martinique, Fort-de-France, Martinique

## Abstract

Ingestion of large amounts of cocaine packages is a well-known method for cross-border transportation. Intestinal obstruction and life-threatening sympathomimetic toxidrome including seizures, ventricular dysrhythmia, and cardiac arrest resulting from the rupture of cocaine packages may occur. Here, we report a case of a 34-year-old pregnant woman who had a sudden cardiac arrest while waiting for her bags at Paris-Charles de Gaulle Airport, France. According to the flight attendants, the patient travelled from Brazil and complained of abdominal pain during the flight. After resuscitation, the patient presented sustained tachycardia and convulsions suggesting cocaine overdose caused by body packing. Once admitted to the hospital, laparotomy was performed allowing the extraction of 50 cocaine packages. Cardiac symptoms were attributed to the rupture of five of the packages. Prehospital and emergency physicians need to be aware of the possibility of cocaine overdose by body packing in patients presenting sudden cardiac arrest in airports.

## 1. Introduction

Cocaine is a widely abused illicit drug extracted from the leaves of *Erythroxylum coca*, native to western South America. The production, distribution, and sale of cocaine is illegal in most countries as regulated by the Single Convention on Narcotic Drugs (1961) [1] and the United Nations Convention Against Illicit Traffic in Narcotic Drugs and Psychotropic Substances (1988) [2]. Thus, several methods of drug smuggling are used to allow cross-border transportation of cocaine including hiding the goods in vehicles, carrying items, attaching them to the body, or using the body as container. The term “Body packing” designates the concealment of illicit drugs in the body, mainly in the gastrointestinal tract for transportation of cocaine, heroin, amphetamines, and cannabinoids [3, 4]. Here, we report a case of sudden cardiac arrest in the airport revealing cocaine body packing and requesting immediate medical and surgical intervention.

## 2. Case Presentation

When attending a flight in correspondence to Spain in Paris Charles de Gaulle international airport, a 34-year-old woman became agitated and subsequently convulsed. Airport medical services were called. According to the flight attendants, the patient was coming from Brazil and complained of abdominal pain during the flight. Initial examination showed Glasgow Coma Score of 6, blood pressure of 175/104 mmHg, heart rate of 136/min, and SpO_2_ of 93% while breathing air. The patient presented general seizures, bilateral mydriasis, and intense sweat. Suddenly, cardiac arrest occurred. The patient was successfully resuscitated by the medical prehospital emergency team and immediately referred to our medical intensive care unit (ICU).

**Figure 1:**
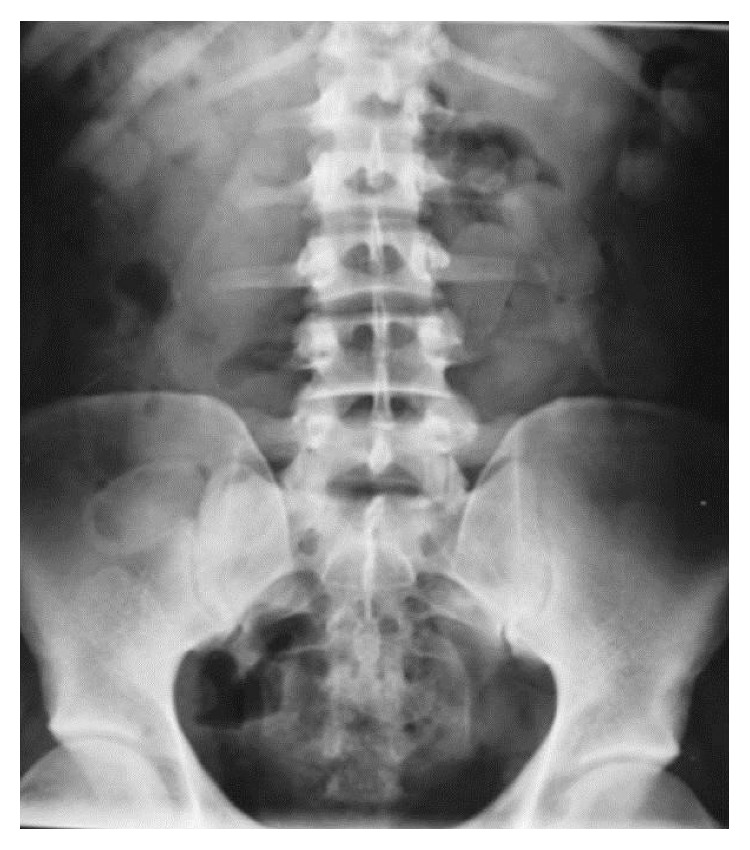
Drug smuggling by body packing in a patient presenting sudden cardiac arrest in the airport.

On ICU admission, the patient was relatively stable. She was intubated and mechanically ventilated. Her blood pressure was 100/62 mmHg and heart rate was 113/min. Physical examination was normal except limited crepitation at pulmonary auscultation. Routine chemistry tests showed sodium 162 mmol/L, potassium 3.6 mmol/L, creatinine 116 *µ*mol/L, bicarbonate 10.5 mmol/L, and and lactate 18.3 mmol/L. Serum creatine kinase was 284 IU/L and troponin I was 5 *µ*g/L. Electrocardiogram revealed irregular tachycardia with enlarged 0.130 s QRS complex. Pregnancy screening was positive but the exact term of pregnancy was unknown although estimated to be in the first trimester. Urine toxicological screening was positive for cocaine. Given the patient's medical history and presentation with abdominal pain, sustained sympathomimetic syndrome and intraventricular block on the electrocardiogram, cocaine body packing was suspected and abdominal plain X-ray performed, showing multiple bags in the gastrointestinal tract (Figure 1).

Rapidly after ICU admission, her cardiovascular situation worsened with typical rapid ventricular tachycardia onset accompanied by a decrease in blood pressure. The patient was transferred to the operating room and immediate laparotomy was performed, allowing the extraction of 50 packets of cocaine in which five were ruptured. Therapeutic hypothermia at 33°C was performed using cold blankets and ice packs during 24 hours. Rapidly after weaning sedation, the patient's conditions improved. Epinephrine was withdrawn. The patient was extubated 24 h postsurgical. ICU outcome was uneventful except for hospital-acquired pneumonia. The patient was discharged on day 4 without neurological sequelae. A few months later, she gave birth to a healthy baby.

## 3. Discussion

Incidence of cocaine body packing from South America to the US and Europe is increasing, attributed to the extension of large international illicit drug trade. The drug is usually placed in packets containing 3–15 g cocaine and made of several layers of latex and a hard wax coating of varying quality [3].

Four types of drug packages have been described including *type-1 packages*, containing loosely packed drug covered with two to four layers of wrapping like condoms and thus at high risk of leakage or rupture; *type-2 packages*, consisting of a bundle of tightly packed drug covered with five to seven layers of tubular latex, each layer having the consistency of a latex glove; *type-3 packages*, presenting as hard drug packages wrapped in aluminum foil and overwrapped with three to five layers of tubular latex securely tied at both ends, obtained by a mechanical manufacturing process; and *type-4 packages*, prepared by dissolving the drug in an alcohol-water solution, then hardened and put in tubular latex completed with colored paraffin or fiberglass to reduce the radiodensity and minimize the risk of detection [5].

Usually, the vast majority of the body packers apprehended by the airport authorities do not require hospitalization [6]. Diagnosis in clinically asymptomatic passengers is based on information obtained by the police, customs agents, or aircraft team members, secondary confirmed by clinical examination and plain abdominal radiographs or CT-scans of the abdomen and pelvis to evidence the presence of foreign bodies. Drug screening in urine is generally negative and thus not useful. However, various issues exist with radiological examinations performed to obtain the diagnosis in suspected body packers. The legislative framework regarding the use of imaging techniques without medical indication vary from one country to another [7]. In some countries like Hong Kong, informed consent must be obtained prior to the radiological examination, whereas in other countries like France or the UK, informed consent is not required and radiological examinations may be performed at the request of a customs officer or following a judicial injunction. Additionally, many drug-trafficking organizations use different materials like aluminum foil, plastic food wrap, carbon paper, and cigarette paper to reduce the radiological detectability; as a result, many cases of false-negative X-ray and CT findings have been described in the literature [8, 9].

Abdominal X-ray is not the gold standard for body packing diagnosis due to low sensitivity and the impossibility to count the exact number of packages. In this case, at least one noncontrasted abdominal CT would have been mandatory to count the exact number of package, even after the operation since not done before due to her life-threatening situation, to ensure that all packages were successfully removed. Interestingly, the size of the packages can widely vary in a single patient, and the total extraction of the package cannot be verified manually and even surgically. However, in this pregnant patient, no additional radiological examination was performed, and clinical monitoring was only decided to ensure package elimination.

Hopefully, complications in cocaine body packers remain relatively rare. Conformingly, in a series of 581 body packers cases referred to a medico-judiciary emergency unit in Paris over a 4-year period, eight subjects developed a complication requiring ICU admission: six acute cocaine intoxications due to packet rupture and two intestinal occlusions [10]. No patient died, and surgical treatment was necessary in six patients. In a survey of Jamaican hospitals focusing at patients who required surgery for cocaine body packing, seventeen patients were identified with 11 cases of bowel obstruction, two of delayed passage of pellets, three of ruptured pellets with cocaine toxicity, and one patient panicked and requested surgery as agitation was considered by the physicians in charge as possibly resulting from cocaine poisoning [11]. In a more recent study reporting 132 cases of body packing, body stuffing and mixed cases, referred by the Swiss authorities to the emergency department over 12 years, a surgical intervention was required in only three body packers (2.3%), owing to stasis of the packages inside the stomach, supporting that medical management of these patients is rarely associated with serious complications [12].

Cocaine acts by adrenergic stimulation both centrally and peripherally by inhibiting the reuptake of norepinephrine and dopamine at the preganglionic sympathetic nerves [13]. Additionally, due to its powerful anesthetic effects blocking the sodium and potassium channels, cocaine exposure may be responsible for direct cardiovascular toxicity. Cocaine alters intraventricular conduction reflected in QRS widening and QT prolongation on the ECG resulting in sustained ventricular arrhythmia and cardiac arrest.

Therefore, severe complications due to cocaine package rupture may occur leading to sudden cardiac arrest like in our case, requiring immediate initiation of cardiopulmonary resuscitation to be continued until the patient is successfully resuscitated. In patients who undergo prolonged external cardiac massage for cocaine-induced cardiac arrest or who develop fulminant heart failure, venoarterial extracorporeal membrane oxygenation (ECMO) should be considered as a lifesaving bridge to recovery [14]. Once admitted to the ICU, targeted temperature control or therapeutic hypothermia is also essential to improve the final neurological prognosis of the comatose cardiac arrest patients like ours [15].

In conclusion, awareness of predisposing factors as well as appropriate use of imaging techniques in the airport is crucial for the establishment of early diagnosis. Once diagnosed, close and careful monitoring of body-packing patients by the prehospital emergency medical service is the key for good prognosis.
